# Assessment of the potential vaping-related exposure to carbonyls and epoxides using stable isotope-labeled precursors in the e-liquid

**DOI:** 10.1007/s00204-021-03097-x

**Published:** 2021-06-22

**Authors:** Anne Landmesser, Max Scherer, Gerhard Scherer, Mohamadi Sarkar, Jeffery S. Edmiston, Reinhard Niessner, Nikola Pluym

**Affiliations:** 1 ABF Analytisch-Biologisches Forschungslabor GmbH, Semmelweisstrasse 5, 82152 Planegg, Germany; 2 Altria Client Services LLC, Center for Research and Technology, Richmond, VA USA; 3 Chair for Analytical Chemistry, Technische Universität München, Marchioninistraße, Munich, Germany

**Keywords:** Electronic cigarettes, Biomarkers of exposure, Carbonyls, Epoxides, Stable isotope-labeled constituents, Mercapturic acids

## Abstract

The formation of carbonyls and epoxides in e-cigarette (EC) aerosol is possible due to heating of the liquid constituents. However, high background levels of these compounds have inhibited a clear assessment of exposure during use of ECs. An EC containing an e-liquid replaced with 10% of ^13^C-labeled propylene glycol and glycerol was used in a controlled use clinical study with 20 EC users. In addition, five smokers smoked cigarettes spiked with the described e-liquid. Seven carbonyls (formaldehyde, acetaldehyde, acrolein, acetone, crotonaldehyde, methacrolein, propionaldehyde) were measured in the aerosol and the mainstream smoke. Corresponding biomarkers of exposure were determined in the user’s urine samples. ^13^C-labeled formaldehyde, acetaldehyde and acrolein were found in EC aerosol, while all seven labeled carbonyls were detected in smoke. The labeled biomarkers of exposure to formaldehyde (^13^C-thiazolidine carboxylic acid and ^13^C-*N*-(1,3-thiazolidine-4-carbonyl)glycine), acrolein (^13^C_3_-3-hydroxypropylmercapturic acid) and glycidol (^13^C_3_-dihydroxypropylmercapturic acid) were present in the urine of vapers indicating an EC use-specific exposure to these toxicants. However, other sources than vaping contribute to a much higher extent by several orders of magnitude to the overall exposure of these toxicants. Comparing data for the native (unlabeled) and the labeled (exposure-specific) biomarkers revealed vaping as a minor source of user’s exposure to these toxicants while other carbonyls and epoxides were not detectable in the EC aerosol.

**Supplementary Information:**

The online version contains supplementary material available at 10.1007/s00204-021-03097-x.

## Introduction

The use of e-cigarettes (ECs) has increased over the past several years (Beard et al. 2020; Cullen et al. 2019; Dai and Leventhal 2019; Kapan et al. 2020). As use of ECs become more prevalent, it becomes more important to understand the potential exposure to harmful chemicals during use of these products. One area of focus is the potential formation of carbonyls, epoxides, and aromatic amines by thermal degradation of e-liquid constituents, despite the much lower temperatures during vaping (< 350 °C) compared to conventional smoking (up to 900 °C) (Farsalinos et al. 2015b; Gillman et al. 2016; Hutzler et al. 2014; Tayyarah and Long 2014) and the resulting exposure to those toxicants by EC vaping.

**Fig. 1:**
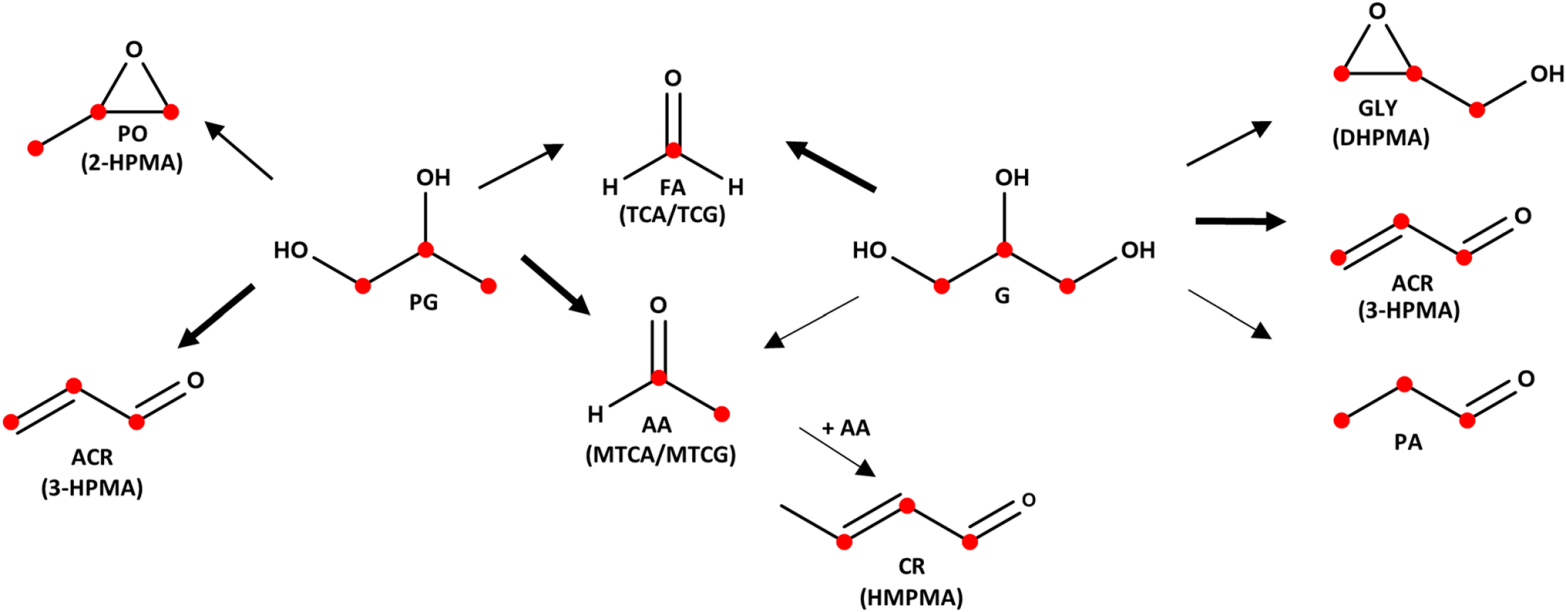
Overview of the thermal degradation products formed from propylene glycol (PG) and glycerol (G). The stable isotope-labeled ^13^C-atoms are illustrated as red dots in the structures. The thickness of the arrows represent the contribution of PG and G to the formation of the degradation products formaldehyde (FA), acetaldehyde (AA), acrolein (ACR), propionaldehyde (PA), crotonaldehyde (CR), glycidol (GLY), and propylene oxide (PO) according to Sleiman et al. (2016) Uchiyama et al. (2020) and our own findings. The corresponding biomarkers ((methyl-)thiazolidine carboxylic acid ((M)TCA), (methyl-)thiazolidine carbonyl glycine ((M)TCG), 2,3-dihydroxypropylmercapturic acid (DHPMA), 3-hydroxypropylmercapturic acid (3-HPMA), 2-hydroxypropylmercapturic acid (2-HPMA), hydroxymethylpropylmercapturic acid (HMPMA) are shown in brackets

The major constituents of e-liquids are propylene glycol (PG), glycerol (G), nicotine, water and flavors. The aerosol is formed by heating the e-liquid and during this process PG and G may be decomposed into toxicants such as the carbonyls formaldehyde (FA), acetaldehyde (AA), acrolein (ACR), and crotonaldehyde (CR) as well as the epoxides glycidol (GLY) or propylene oxide (PO), to which vapers might be exposed (Fig. 1) (Flora et al. 2016; Sleiman et al. 2016; Sodhi and Khanna 2015; Uchiyama et al. 2020).

The aforementioned carbonyls and epoxides are found in the environment, in food, as well as possibly formed endogenously (Bakhiya et al. 2011; Feron et al. 1991; Moghe et al. 2015). Hence, identifying the EC-specific contribution to carbonyl and epoxide exposure is challenging. In the case of ECs, this problem can be circumvented using stable isotope-labeled PG and G in the e-liquid. Studies using stable isotope tracers have long been used in mass spectrometry as the “gold” standard method for understanding kinetics, uptake, distribution, metabolism and elimination of various compounds in living organisms (Bequette et al. 2006; Darmaun and Mauras 2005; Evershed et al. 2006; Wittmann 2002).

**Fig. 2:**
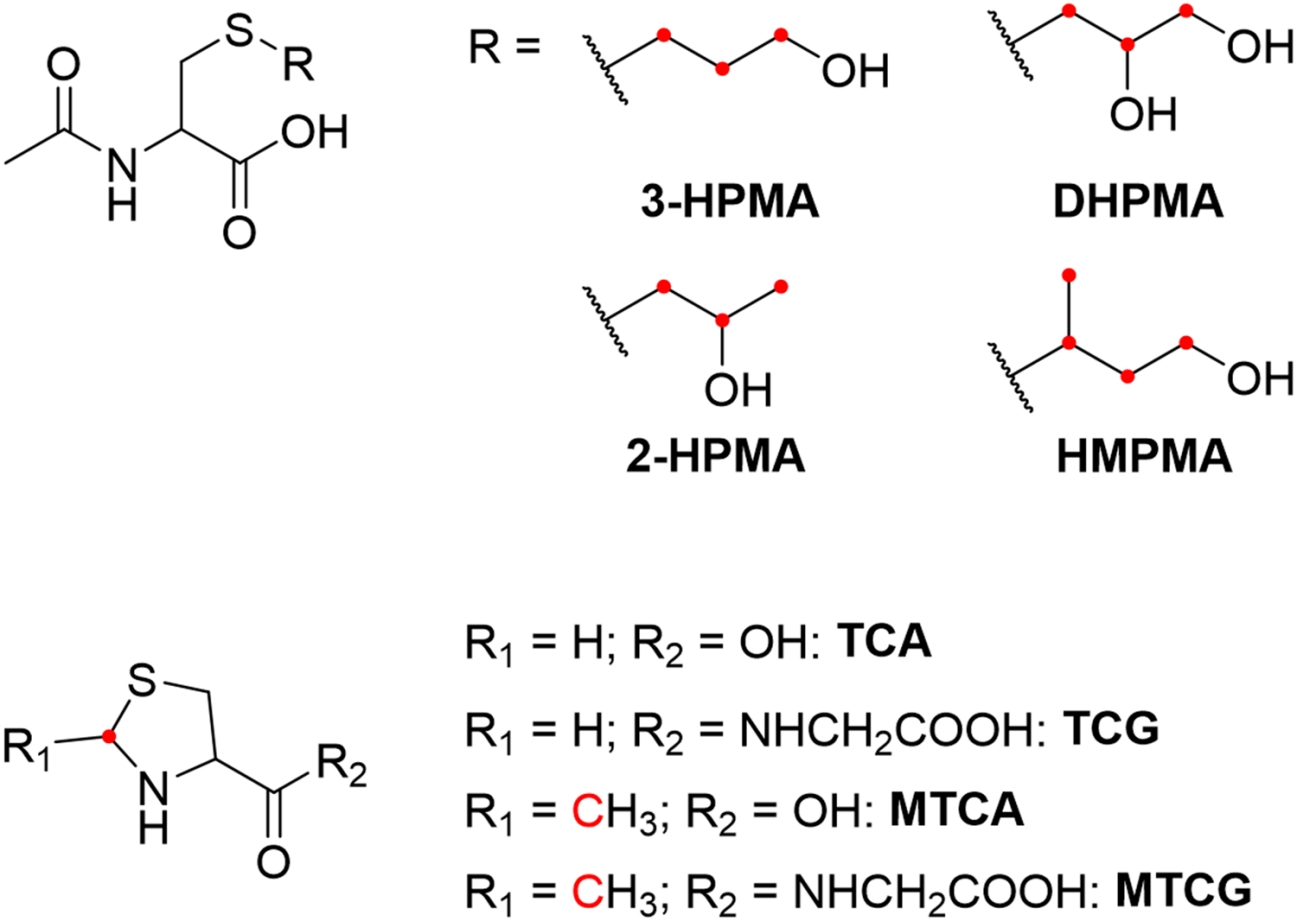
Structures of the measured metabolites. The stable isotope-labeled ^13^C-atoms are illustrated as red dots in the structures. The names of the compounds are listed in Fig. 1

Our approach comprised of the analysis of carbonyls and epoxides in the aerosol derived from ECs containing e-liquids in which 10% of PG, G and nicotine were replaced by stable isotope-labeled analogues, as well as the determination of corresponding biomarkers in the urine of vapers who used these ECs in a clinical study under controlled but realistic vaping conditions. We hypothesized that the compounds which were predominantly formed by degradation of PG and G should be present at a ratio of approx. 10:1 between the native (unlabeled) and the labeled form in the aerosol. Higher ratios would indicate other sources than PG and G for their formation during aerosol generation. This ratio can give an estimation with respect to product use-specific uptake by measuring the corresponding biomarkers of exposure in urine, namely thiazolidine carboxylic acid (TCA) and *N*-(1,3-thiazolidine-4-carbonyl)glycine (TCG) for FA, methyl-thiazolidine carboxylic acid (MTCA) and *N*-(2-methyl-1,3-thiazolidine-4-carbonyl)glycine (MTCG) for AA, 2,3-dihydroxypropylmercapturic acid (DHPMA) for GLY, 3-hydroxypropylmercapturic acid (3-HPMA) for ACR, 3-hydroxy-1-methylpropylmercapturic acid (HMPMA) for CR, and 2-hydroxypropylmercapturic acid (2-HPMA) for PO (Fig. 2). Smokers using cigarettes spiked with the labeled PG, G and nicotine were also included as ‘positive control’ in the clinical study. It was assumed that at the high temperature of tobacco combustion the labeled carbonyls and epoxides are formed and can be detected in mainstream smoke (Diekmann et al. 2006; Sampson et al. 2014) as well as the corresponding labeled biomarkers in the smokers’ urine.

## Materials and methods

### Clinical Study

The clinical study has been described in detail earlier (Landmesser et al. 2019). Briefly, 25 healthy adult males belonging to three groups participated in the study: 5 regular smokers (smoking non-filter cigarettes spiked with 13.4 mg ^13^C_3_-PG, 13.6 mg ^13^C_3_-G, and 2.4 mg D_7_-nicotine dissolved in 100 µL ethanol per cigarette), 10 regular vapers (vaping-labeled e-liquid at 10 W) and 10 regular vapers (vaping-labeled e-liquid at 18 W). In the applied e-liquid containing PG and G 50/50% (m/m) and 1.2% nicotine (m/m), 10% of each PG, G and nicotine was replaced by ^13^C_3_-PG, ^13^C_3_-G and D_7_-nicotine, respectively. The e-liquid was tested with respect to the presence of the (un)labeled carbonyls formaldehyde, acetaldehyde, acrolein, acetone, crotonaldehyde, methacrolein, and propionaldehyde. No carbonyls were detectable in the e-liquid. The labeled compounds were purchased from AptoChem, Montreal, Canada. All three substances were characterized for their identity, chromatographic purity, water content, and residual solvents. ^13^C_3_-PG, ^13^C_3_-G, and D_7_-nicotine showed a purity of 99.2%, 100%, and 99.7%, respectively. The subjects stayed in the clinic for 84 h (evening of day-1 until morning of Day 4). Vaping or smoking took place only on day 1 during 10 sessions, each comprising the consumption of 10 controlled puffs (two puffs per minute, 4 s puff duration) by the 20 vapers or one cigarette by the 5 smokers. Before and after each sampling in the clinical study, the tanks were weighed to determine the amount of e-liquid consumed per session. The amount of e-liquid consumed was used to normalize the results. All urine voids were collected from the morning of Day 1 prior to the first vaping/smoking session until the morning of day 4.

### Chemicals, standards and stock solution

Acetaldehyde-dinitrophenylhydrazone (DNPH) (99.9% purity), acetone-DNPH (99.7%) acrolein-DNPH (99.8%), formaldehyde-DNPH (99.9%), crotonaldehyde-DNPH (99.6%), methacrolein-DNPH (96.7%) and propionaldehyde-DNPH (98.3%) were purchased from Neochema (Bodenheim, Germany). 3,5,6-D_3_-Acetaldehyde-DNPH (99%), 3,5,6-D_3_-acetone-DNPH (99%), 3,5,6-D_3_-acrolein-DNPH (99%), 3,5,6-D_3_-formaldehyde-DNPH (98.8%), 3,5,6-D_3_-crotonaldehyde-DNPH (99%) and 3,5,6-D_3_-propionaldehyde-DNPH (98.6%) were obtained from CDN Isotopes Inc. (Quebec, Canada). 2,4-Dinitrophenylhydrazine for HPLC derivatization (> 99%), perchloric acid (70%) and pyridine (anhydrous, 99.8%) were purchased from Sigma (Taufkirchen, Germany). Acetonitrile (ULC/MS grade) was obtained from Biosolve BV (Valkenswaad, Netherlands). Ultrapure water was prepared using the arium® pro ultrapure water system (Sartorius, Göttingen, Germany).

### Analysis of carbonyls in aerosol and smoke

The carbonyls (formaldehyde, acetaldehyde, acrolein, acetone, crotonaldehyde, methacrolein, propionaldehyde) are highly volatile and reactive and therefore they were derivatized with 2,4-dinitrophenylhydrazine upon trapping, followed by dilution. 10 puffs of the EC or a single cigarette (approx. 7–8 puffs) were drawn through two glass impingers in sequence each containing 20 mL of an acidic dinitrophenylhydrazine derivatization solution (2.5 mM in acetonitrile) according to Miller et al. (2010). The puffing regime was set according to CORESTA recommended method CRM no. 81 (CORESTA 2015) with minor modifications: puff duration: 4 s (instead of 3 s according to CRM no. 81), puff interval: 30 s, puff volume: 55 mL. Immediately after the trapping procedure, 200 μL pyridine was added to stop the derivatization reaction. The trapping solution was diluted 1:10 (1:100 for the cigarette) with acetonitrile. Prior to the UPLC-MS/MS analysis, 10 μL of the internal standard mix of the carbonyl-DNPHs was added to 100 μL of the diluted sample, 5 μL of which was injected for analysis.

Liquid chromatography was performed with a Shimadzu Nexera X2 UPLC system consisting of a binary pump, an auto-sampler, a degaser, and a column oven (Shimadzu Corp., Kyoto, Japan). A triple quadrupole mass spectrometer QTRAP® 6500 + equipped with a Turbo V ion spray source, operating in negative ESI mode, was used for detection (AB Sciex, Darmstadt,Germany). High purity nitrogen was produced by a nitrogen generator NGM 22-LC/MS (cmc Instruments, Eschborn, Germany). Chromatographic separation was achieved on a Kinetex® 5 μm EVO C18 column (150 × 2.1 mm, 5 μm, Phenomenex, Aschaffenburg, Germany) with water (eluent A) and acetonitrile (eluent B) applying the following gradient: 0–5.0 min: 35–55% B; 5.0–7.0 min: 70% B; 7.0–7.1 min: 70–35% B; 7.1 – 10.0 min: 35% B. The column was kept at 50 °C with a flow rate of 0.7 mL/min. Labeled and unlabeled analytes as well as their corresponding internal standards were monitored in the multiple reaction monitoring mode (MRM, Supplementary Information Table S1).

All determinations of the carbonyls were repeated eight times for EC aerosol at low (10 W) and high (18 W) wattage and ten times for cigarette mainstream smoke, respectively. The presented data were corrected for isotope overlap according to Scherer et al. (2010). The lower limit of quantification (LLOQ) was 0.2 ng/puff for EC aerosol and 0.25 ng/puff for cigarette mainstream smoke. The upper limit of quantification (ULOQ) was 100 ng/puff for EC aerosol and 125 ng/puff for cigarette mainstream smoke.

### Analysis of epoxides in aerosol and mainstream smoke

Various trapping agents and chromatographic conditions were tested for the determination of the epoxides propylene oxide (PO) and glycidol (GLY) in EC aerosol and mainstream smoke. The analytical method showing the best performance with regard to the sensitivity and recovery is presented in the Supplementary Information. However, the sensitivity of the final method with an LLOQ of 0.05 µg/mL was still not sufficient for the quantification of PO and GLY in EC aerosols or smoke in our study.

### Analysis of the biomarkers for formaldehyde and acetaldehyde

The specific biomarkers TCA and TCG for FA as well as MTCA and MTCG for AA were determined according to the fully validated method published by Landmesser et al. (2020). Briefly, the biomarkers were cleaned up from major matrix components by solid-phase extraction followed by derivatization under alkaline conditions using propyl chloroformate. The LC–MS/MS analysis was performed using a Shimadzu Nexera X2 UPLC system consisting of a binary pump, an autosampler, a degaser and a column oven (Shimadzu Corp., Kyoto, Japan) combined with a triple quadrupole mass spectrometer QTRAP® 6500 + equipped with a Turbo V ion spray source (AB Sciex, Darmstadt, Germany), operated in positive ESI (ESI+) mode. The presented data are corrected for isotope overlap according to Scherer et al. (2010). LLOQs and ULOQs were 0.5 ng/mL and 200 ng/mL for (M)TCA and 1.0 ng/mL and 400 ng/mL for (M)TCG in accordance with Landmesser et al. (2020).

### Analysis of mercapturic acids

The following mercapturic acids were determined in 24 h urine samples according to Pluym et al. (2015) with modifications: 3-HPMA (biomarker for acrolein), HMPMA (crotonaldehyde), 2-HPMA (propylene oxide), and DHPMA (glycidol). DHPMA, which was not implemented in the initial method (Pluym et al. 2015) was included for the purpose of our study into the analytical method for 2-/3-HPMA. LLOQs (ng/mL) of the newly integrated analytes (DHPMA and all labeled mercapturic acids) were as follows: [^13^C_4_]-HMPMA: 5.0, [^13^C_3_]-2-HPMA: 0.5, [^13^C_3_]-3-HPMA: 0.5, DHPMA: 10; [^13^C_3_]-DHPMA: 0.8. ULOQs (ng/mL) of the newly integrated analytes were as follows: [^13^C_4_]-HMPMA: 2,500, [^13^C_3_]-2-HPMA: 2,000, [^13^C_3_]-3-HPMA: 10,000, DHPMA: 2,000; [^13^C_3_]-DHPMA: 2,000. The applied mass transitions for the labeled analytes were selected according to the published fragmentation pathways of mercapturic acids (summarized in Supplementary Information Table S2).

Biomarker analysis was performed separately for urine fractions collected over 48 h, beginning with the first fraction voided after start of the first vaping/smoking session on Day 1. The presented data are corrected for isotope overlap according to Scherer et al. (2010).

## Results

### Carbonyl and epoxide levels in mainstream smoke of cigarettes and aerosol of ECs

In cigarette mainstream smoke, all carbonyls expected to be formed from labeled PG and G according to Fig. 1 were detected (labeled FA, AA, ACR, and PA). In addition, ^13^C-labeled CR, AT, and MA were observed. Mean concentrations ranged from 6.3 ng/puff for ^13^C-FA to 290 ng/puff for ^13^C_3_-AT (Table 1). The corresponding unlabeled compounds were obtained at much higher amounts in smoke, mostly well above the ULOQ, and not further evaluated in this study.

**Table 1:** Mean yields (standard deviations) of unlabeled and ^13^C-labeled carbonyls on a per puff basis in smoke and aerosol (ng/puff)

	FA	^13^C-FA	AA	^13^C_2_-AA	ACR	^13^C_3_-ACR	CR	^13^C_2_-CR	AT	^13^C_3_-AT	MA	^13^C_4_-MA	PA	^13^C_3_-PA
Mainstream smoke of cigarettes	73.8 (26.1)	6.34 (2.86)	> > 125^1^	194^2^ (49.5)	> > 125^1^	61.2 (18.2)	> > 125^1^	88.1 (35.8)	> > 125^1^	288^2^ (39.3)	> 125^1^	59.1 (10.9)	> > 125^1^	58.4 (12.5)
EC Aerosol (10 W)	22.6 (8.23)	2.36 (0.76)	18.8 (5.10)	0.23 (0.05)	6.36 (3.43)	0.66 (0.22)	< LLOQ	< LOD	8.66 (4.83)	< LLOQ	< LLOQ	< LOD	1.80 (0.32)	< LOD
EC Aerosol (18 W)	39.1 (8.40)	4.39 (0.63)	45.9 (15.4)	0.55 (0.16)	10.2 (5.98)	1.10 (0.60)	0.27 (0.19)	< LOD	8.66 (4.33)	< LLOQ	< LLOQ	< LOD	3.95 (1.79)	< LOD
*R* in aerosol 10 W	9.6		81		9.6		n.a.^3^		n.a.^3^		n.a.^3^		n.a.^3^	
*R* in aerosol 18 W	8.9		84		9.3		n.a.^3^		n.a.^3^		n.a.^3^		n.a.^3^	

Note that only 10% of the e-liquid were replaced with ^13^C-propylene glycol and ^13^C-glycerol (50:50 (v/v))

*FA* formaldehyde, *AA* acetaldehyde, *ACR* acrolein, *CR* crotonaldehyde, *AT* acetone, *MA* methacrolein, *PA* propionaldehyde, *R* ratio between unlabeled and labeled concentrations in the aerosol, averaged for the amounts received at 10 W and 18 W

^1^Unlabeled carbonyls were much higher (mostly by several orders of magnitude) than carbonyl yields for EC aerosol and not quantitatively evaluated in this study

^2^> ULOQ

^3^Ratio was not calculated since the concentrations of the labeled form were either below the LLOQ or below the LOD: LOD: 0.06 ng/puff (EC aerosol), 0.07 ng/puff (mainstream smoke); LLOQ: 0.2 ng/puff (EC aerosol), 0.25 ng/puff (mainstream smoke); ULOQ: 100 ng/puff (EC aerosol), 125 ng/puff (mainstream smoke)

The observed per puff amounts in the aerosol of the EC were lower than in the cigarette smoke by several orders of magnitude for the labeled carbonyls. The lowest difference was observed for ^13^C-FA with similar concentrations in EC aerosol and smoke. Only ^13^C-FA, ^13^C_2_-AA and ^13^C_3_-ACR were quantifiable in the aerosol, averaging 2.36 and 4.39 ng/puff, 0.23 and 0.55 ng/puff and 0.66 and 1.10 ng/puff for the 10 W and 18 W vaping conditions, respectively. Notably, the increase of these carbonyls in the 18 W compared to the 10 W vaping conditions was around 1.6- to 2.3-fold resembling the increase in the wattage of 1.8-fold. ^13^C-labeled CR, MA and PA were not detectable (below the limit of detection (LOD)) and ^13^C_3_-AT was below the LLOQ in EC aerosol. In analogy to the labeled carbonyls, vaping at 10 W yielded lower amounts than at 18 W for the unlabeled analytes. The following rank order was observed for unlabeled carbonyls: methacrolein (< LLOQ) < crotonaldehyde (< LLOQ at 10 W and 0.27 ng/puff at 18 W) < propionaldehyde < acetone ≈ acrolein < formaldehyde ≈ acetaldehyde (see Table 1).

A ratio (R = amount unlabeled/amount labeled) of around 10 was observed for FA and ACR in aerosol reflecting the 10% replacement of PG and G in the e-liquid by labeled PG and G, while AA yielded a significantly higher ratio of 81–84 (Table 1).

The concentrations of labeled FA, AA, and ACR were expressed as ng/g e-liquid consumed. This normalization takes into account the variability in aerosol generation due to the differing wattage applied. Thus, yields per gram e-liquid consumed should be better suited to compare aerosol amounts EC between studies regardless of the puffing regime applied (Farsalinos and Gillman 2017). There were no significant differences in the yields based on e-liquid consumption between the two power settings with highest concentrations found for labeled FA (150 and 162 ng/g e-liquid) followed by labeled ACR (80 and 80 ng/g e-liquid) and AA (15 and 20 ng/g e-liquid).

### Quantification of labeled and unlabeled biomarkers in urine of smokers and vapers

**Fig. 3:**
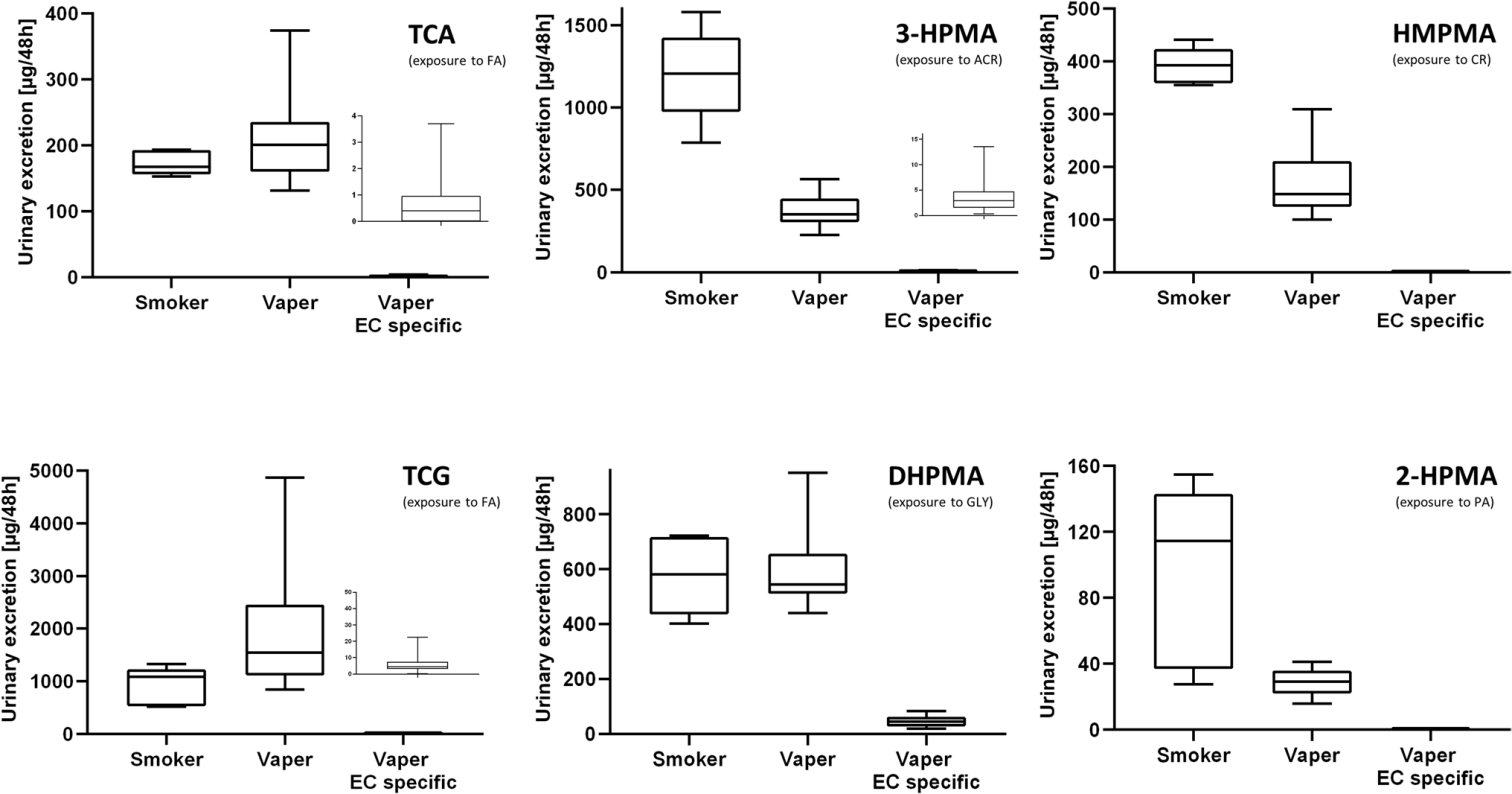
Box plots of the total amounts of the biomarkers excreted over 48 h after controlled vaping/smoking. Total amounts were calculated as the sum of labeled and unlabeled for smokers (left box plot) and vapers (middle box plot). The EC-specific exposure corresponds to the excreted amount of labeled biomarker multiplied by 10 to account for the 10% replacement (right box plot: Vaper EC-specific). The inserted figures in the graphs for TCA, TCG and 3-HPMA show the box plots for the vaping-specific excretion with y-axis magnified by 100-fold for better illustration. Lines and boxes represent the median and the 25th/75th percentile, respectively. Error bars illustrate the 5th and 95th percentile. *TCA/TCG* biomarkers of formaldehyde, *DHPMA* biomarker of glycidol, *3-HPMA* biomarker of acrolein, *2-HPMA* biomarker of propylene oxide, *HMPMA* biomarker of crotonaldehyde

The amounts of labeled and unlabeled biomarkers excreted over 48 h after controlled vaping or smoking are summarized in Table 2. While ^13^C_3_-2-HPMA (formed from inhaled ^13^C_3_-PO) was only found in smokers, ^13^C_3_-3-HPMA (^13^C_3_-ACR), ^13^C_3_-DHPMA (^13^C_3_-GLY) and ^13^C-TCA/^13^C-TCG (^13^C-FA) were observed in quantifiable amounts both for smokers and vapers (Fig. 3). The mercapturic acids ^13^C_2/4_-HMPMA (^13^C_2/4_-CR) and ^13^C_2_-MTCA/^13^C_2_-MTCG (^13^C_2_-AA) were not detected, neither in smokers nor in EC vapers.

**Table 2:** Mean (SD) amounts excreted in urine of smokers and EC users (vapers) over 48 h (µg) of the unlabeled and ^13^C-labeled biomarkers of exposure

	TCA	^13^C-TCA	TCG	^13^C-TCG	MTCA	^13^C_2_-MTCA	MTCG	^13^C_2_-MTCG	3-HPMA	^13^C_3_-3-HPMA	DHPMA	^13^C_3_-DHPMA	2-HPMA	^13^C_3_-2-HPMA	HMPMA	^13^C_2/4_-HMPMA
Smoker	171.3 (16.5)	1.56 (0.55)	912.8 (328.3)	6.31 (2.97)	40.3 (20.1)	< LOD	15.2 (6.9)	< LOD	1057 (221.1)	143.2 (32.8)	562.0 (122.6)	15.8 (7.78)	51.6 (17.2)	43.2 (33.6)	391.6 (31.1)	< LOD
Vaper 10 W	198.3 (44.7)	0.12 (0.14)	2039 (1158)	0.60 (0.58)	40.4 (9.0)	< LOD	17.9 (4.2)	< LOD	381.7 (91.8)	0.47 (0.39)	635.1 (141.1)	4.59 (1.35)	32.6 (6.7)	< LOD	150.7 (49.7)	< LOD
Vaper 18 W	218.5 (67.6)	0.03 (0.04)	1562 (579.9)	0.66 (0.58)	46.2 (16.5)	< LOD	15.6 (4.2)	< LOD	368.9 (101.0)	0.28 (0.19)	529.4 (69.6)	4.74 (2.36)	26.5 (7.0)	< LOD	191.9 (56.8)	< LOD
*R* in vapers 10 W	1600		3400		n.a		n.a		810		140		n.a		n.a	
*R* in vapers 18 W	6500		2400		n.a		n.a		1300		110		n.a		n.a	

For details on product use, see materials and methods and our previous publication(Landmesser et al. 2019)

*TCA/TCG* metabolites of FA, *MTCA/MTCG* metabolites of AA, *3-HPMA* metabolite of ACR, *DHPMA* metabolite of GLY, *2-HPMA* metabolite of PO, *HMPMA* metabolite of CR, *R* ratio between unlabeled and labeled biomarker in urine of vapers

For labeled 3-HPMA and 2-HPMA, smokers excreted at least 2–3 orders of magnitude higher amounts than the EC vapers. This difference was less pronounced (only 3–20-fold) in the case of labeled DHPMA and TCA/TCG, respectively (Fig. 3). Labeled 3-HPMA, DHPMA, TCA, and TCG were obtained at similar concentrations for the two vaping conditions (10 and 18 W).

In terms of the unlabeled compounds, all biomarkers of interest were measurable in each subject. The ratios between unlabeled and labeled biomarkers in vapers ranged from 110 to 6500 (Table 2). The vaping-specific excretion of the biomarkers was calculated by multiplying the amount of labeled biomarker with the factor 10 (considering the 10% replacement with labeled PG and G in the e-liquid). These amounts are illustrated in Fig. 3 (Vaper EC specific).

The unlabeled biomarkers 3-HPMA, 2-HPMA, and HMPMA tended to be higher in smokers, whereas TCA, TCG, MTCA, MTCG and DHPMA showed similar levels in smokers and vapers (Table 2).

## Discussion

Our data unequivocally confirm the formation of FA, AA, and ACR from the precursors PG and G in e-liquid during vaping, which is in line with previous studies (Farsalinos et al. 2015a; Geiss et al. 2015; Goniewicz et al. 2014; Sleiman et al. 2016). Geiss et al. who used a second-generation atomizer device similar to the EC used in our study, found comparable levels of FA, AA, and ACR (Geiss et al. 2015), while most studies reported higher aldehyde concentrations which may result from the different device characteristics and varying puffing regimes used throughout the studies as discussed in detail by Farsalinos et al. (2017). Interestingly, the higher carbonyl concentrations per puff at 18 W are clearly related to increased aerosol generation as can be deduced from the fact that the amounts of carbonyls per mass consumed e-liquid were found to be identical for both wattages. This observation indicates that, at least under the vaping conditions we applied, increasing the power from 10 to 18 W elevated the amount of aerosol produced but not the percentage of PG and G which is converted to carbonyls.

The introduction of labeled PG and G allowed us to assess the formation of carbonyls specifically formed from these e-liquid constituents. The ratio of the unlabeled to the ^13^C-labeled PG and G in the e-liquid of 10:1 was well reflected in the EC aerosol for FA and ACR (Table 1), indicating that both aldehydes are mainly (or almost exclusively) formed from PG and G during vaping. In contrast, for AA a ratio of around 80 was observed, while CR, AT and PA were only detected in their unlabeled form. This suggests that other sources than PG and G contribute to a major extent to the formation of AA. Sugars and flavors were recently discussed as possible sources for carbonyl emissions in EC aerosols (Fagan et al. 2018; Khlystov and Samburova 2016). The use of stable isotope-labeled flavoring ingredients and sugars could be a useful strategy in future studies to further evaluate their chemical fate with regard to vaping.

All seven ^13^C-labeled aldehydes investigated in our study were found in cigarette mainstream smoke indicating that the formation of AA, CR, AT, and PA from PG and G is possible, albeit at higher temperatures than achievable under common vaping conditions (up to 350 °C) (Geiss et al. 2016). Apparently, AA, AT and PA (C2 and C3 compounds) are formed due to decomposition of PG and G (Uchiyama et al. 2020). CR (4 carbon atoms) is more likely to be formed after decomposition of PG and G from AA by aldol condensation (Fig. 1) implying the need for harsher conditions—in this case higher temperatures (Luo and Falconer 1999).

Labeled biomarker analysis in urine allowed us to link the machine-derived aerosol data with actual human exposure values. The difference in smoke and vapor yields was clearly reflected in the biomarker levels for FA and ACR since the respective labeled biomarkers ^13^C-TCA, ^13^C-TCG and ^13^C_3_-3-HPMA were detected at much higher levels in smokers (Fig. 3). Hence, our approach using stable isotope-labeled precursors was capable of detecting exposure to FA and ACR specifically from vaping at a daily intake of approx. 1.1 to 2.2 µg based on the aerosol data and the average e-liquid consumption of 1.2 g per day (Landmesser et al. 2019). Unfortunately, the labeled biomarkers of AA exposure, MTCA and MTCG, respectively, could not be determined due to insufficient long-term stability in urine of only 2 months (Landmesser et al. 2020). Thus, MTCA and MTCG are not suited to assess AA exposure from smoking and vaping.

Furthermore, it was assumed that PG and G may also serve as precursors for the epoxides PO and GLY (Uchiyama et al. 2020). For methodological problems, the expected (labeled) epoxides could not be determined, neither in mainstream smoke of conventional cigarettes nor in aerosol of ECs. Analysis of the corresponding labeled biomarkers in urine revealed exposure to PO (2-HPMA) exclusively in smokers while glycidol exposure (DHPMA) was detected both in the case of smoking and vaping, at approx. threefold lower amounts in the EC vapers (Table 2). Thus, PO seemed to be formed from the precursors PG and G under pyrolytic (smoking) conditions only, while GLY was observed at appreciable amounts already under vaping conditions as can be concluded from the biomarker analysis. Moreover, there was no difference in biomarker levels between subjects using the EC at 10 and 18 W despite the higher per puff carbonyl yields in the aerosol at 18 W. In contrast, excretion rates of PG in urine over 48 h correlated with the increased wattage (Landmesser et al. 2019). Presumably, the generally low exposure to carbonyls in our study was not sufficient to discriminate the vaper subgroups in terms of biomarker excretion rates. These findings emphasize the importance of biomarker analysis in addition to machine-derived aerosol data for a comprehensive exposure and risk assessment.

This study demonstrates the potential utility of the stable isotope labeling approach by comparing biomarker data for unlabeled and labeled compounds from cigarette smoke and EC aerosols. For the unlabeled biomarkers, differences between smokers and vapers were marginal (if any) for TCA/TCG (exposure to FA) and DHPMA (GLY). A more pronounced difference for the unlabeled biomarkers was obtained in the case of PO (2-HPMA), ACR (3-HPMA), and CR (HMPMA) since smoking of combustible cigarettes is a major source as reported in previous studies (Alwis et al. 2012, 2015; Pluym et al. 2015).

The difference in the ratio between the unlabeled and labeled biomarkers of exposure was much more pronounced (*R* = 110–6500) compared with the ratios found for aerosol (9–84) (Table 2). Moreover, the estimated EC-specific uptake in vapers (box plot termed “Vaper EC specific” in Fig. 3) compared to the overall exposure in vapers and smokers (box plots termed “Vaper” and “Smoker” in Fig. 3) demonstrated that EC use only accounted for approximately 0.4%, 1.0%, and 8% of the overall exposure to formaldehyde (TCA/TCG), acrolein (3-HPMA) and glycidol (DHPMA) in vapers (see Supplementary Information for details about the calculation) while no EC specific uptake of PO (2-HPMA) and CR (HMPMA) was observed. This indicates other sources than vaping, like diet and endogenous formation, contribute primarily to the observed biomarker levels of TCA/TCG, 3-HPMA and DHPMA in exclusive vapers (Cederbaum 2012; Cloos et al. 2008; de Groot et al. 2009; Dhareshwar and Stella 2008; Eckert et al. 2011; Hou and Yu 2010; O'Sullivan et al. 2004). For example, vegetable oils and fats were recently identified as a major source of glycidol exposure (Abraham et al. 2019).

The results of this study should be considered in the context of the limitations with respect to the investigated specimen and the sample size in this study. In addition to the aerosol analysis and urinary excretion discussed here, local effects in terms of toxicant exposure may not be neglected as for example nicotine was identified as a source of NNN exposure in the oral cavity of EC vapers (Bustamante et al. 2018). Local exposure, e.g., in the oral cavity was beyond the scope of our study. However, possible local effects should be addressed in future studies for a more profound risk assessment in terms of EC use. Regarding the sample size, 20 male vapers were included limiting the generalizability of the results. Moreover, thermal degradation of PG and G depends on the applied power settings and properties of the device (Gillman et al. 2016). Hence, larger studies including both sexes and users of different EC devices are needed to substantiate our findings. Nevertheless, our study clearly demonstrates the advantage of the stable isotope labelling approach to decipher the EC use specific exposure to toxicants which may be formed from the main constituents PG and G.

## Conclusions

We were able to determine the exposure to various carbonyls and epoxides, resulting from thermal degradation of propylene glycol and glycerol under smoking and vaping conditions using a stable isotope labeling approach. The formation of several toxicants such as crotonaldehyde, methacrolein and acetone were only observed during combustion of conventional cigarettes while propylene glycol and glycerol can also be decomposed to formaldehyde, acetaldehyde, acrolein and glycidol under common vaping conditions, overall at a much lower degree compared to smoking. Assessment of the corresponding biomarkers of exposure in urine confirmed the findings from aerosol data for formaldehyde and acrolein, while a suitable biomarker for acetaldehyde exposure still needs to be identified. This is the first study to prove the EC-specific uptake of glycidol due to the degradation of propylene glycol and/or glycerol during vaping. However, vaping appears to be a minor source with respect to the general exposure to formaldehyde, acrolein and glycidol.

## Supplementary Information

Below is the link to the electronic supplementary material.

Supplementary file1 (DOCX 19 KB)

## Data Availability

Not applicable.
