# Supplementary material for: Assessment of the potential vaping-related exposure to carbonyls and epoxides using stable isotope-labeled precursors in the e-liquid
Source: Arch Toxicol. 2021 Jun 22;95(8):2667–76. doi: 10.1007/s00204-021-03097-x (PMC8298337; doi:10.1007/s00204-021-03097-x)
Supplement: Supplementary file 1 — Supplementary file1 (DOCX 19 KB) [file 204_2021_3097_MOESM1_ESM.docx]

**Supplementary Material to the manuscript:**

Assessment of the potential vaping-related exposure to carbonyls and epoxides by using stable isotope-labeled precursors in the e-liquid

Anne Landmesser^1,3,^ Max Scherer^1^; Gerhard Scherer^1^; Mohamadi Sarkar^2^; Jeffery S Edmiston^2^; Reinhard Niessner^3^; Nikola Pluym^1*^

^1^ABF Analytisch-Biologisches Forschungslabor GmbH, Semmelweisstrasse 5, 82152 Planegg, Germany

^2^Altria Client Services LLC. Center for Research and Technology, Richmond, Virginia, USA

^3^ Chair for Analytical Chemistry, Technische Universität München, Marchioninistraße, Munich, Germany

For submission to: *Archives of Toxicology*

Original Article

*Corresponding author: Dr Nikola Pluym, e-mail: [nikola.pluym@abf-lab.com](mailto:nikola.pluym@abf-lab.com)

### **Analysis of epoxides in aerosol and smoke**

Normalization for e-liquid consumption and puffing regime were performed as described for the analysis of carbonyls in aerosol and smoke. Aerosol and smoke, respectively, were drawn through two glass impingers. Each impinger contained 10 mL of 1 µg/mL D_6_-propylene oxide in toluene. After collection, 2 *μ*L of each impinger were injected into the GC-MS system.

GC-MS was performed with an ISQ in conjunction to a Trace GC Ultra (Thermo Fisher Scientific, MA, US). The single quadrupole mass spectrometer was operated in EI mode. Chromatographic separation was achieved on a PoraBOND Q (25 m x 0.25 mm, 0.25 µm; Agilent, Waldbronn, Germany) with helium as carrier gas. The injector temperature was set to 250 °C. 2 *µ*L of the sample was injected with a split ratio of 1:20. The initial temperature was held for 2 minutes and increased by a rate of 15°C/min to 250 °C. The carrier flow was set to 1.0 mL/min. The analytes were monitored in the selected ion monitoring mode with the following target ions: PO: m/z 43, m/z 58; D_6_-PO: m/z 64; GLY: m/z 43, m/z 44. The calibration ranged from 0.05 µg/mL to 20 µg/mL.

PO (99.9%), and GLY (99.7 %) were purchased from Sigma Aldrich. D_6_-PO (98 %) was purchased from Polymersource (Quebec, Canada).

**Detection parameters for the analysis of carbonyls**

Table S1: MS/MS-parameters for the labeled and unlabeled carbonyl-DNPH-derivatives and their corresponding internal standards sorted by retention time (IS: internal standard, MRM: multiple reaction monitoring)

| Analyte | Quantifier MRM [m/z → m/z] | Qualifier MRM [m/z → m/z] | IS | IS MRM  [m/z → m/z] |
| --- | --- | --- | --- | --- |
| Formaldehyde  ^13^C-Formaldehyde | 209 → 163  210 → 164 | 209 → 151  210 → 151 | D_3_-Formaldehyde | 212 → 166 |
| Acetaldehyde  ^13^C_2_-Acetaldehyde | 223 → 163  225 → 164 | 223 → 151  225 → 151 | D_3_-Acetaldehyde | 226 → 166 |
| Acrolein  ^13^C_3_-Acrolein | 235 → 163  238 → 164 | 235 → 158  238 → 158 | D_3_-Acrolein | 238 → 166 |
| Propionaldehyde  ^13^C_3_-Propionaldehyde | 237 → 163  240 → 164 | 237 → 179  240 → 180 | D_3_-Propionaldehyde | 240 → 166 |
| Crotonaldehyde  ^13^C_2_-Crotonaldehyde  ^13^C_4_-Crotonaldehyde | 249 → 172  251 → 173  253 → 173 | 249 → 151  251 → 151  253 → 151 | D_3_-Crotonaldehyde | 252→ 175 |

**Detection parameters for the analysis of mercapturic acids**

Table S2: Mass transitions of labeled mercapturic acids and their internal standards (IS)

| Method | Analyte | Quantifier MRM [m/z → m/z] | Qualifier MRM [m/z → m/z] | IS | IS MRM  [m/z → m/z] |
| --- | --- | --- | --- | --- | --- |
| MA I | HMPMA  ^13^C_2_-HMPMA  ^13^C_4_-HMPMA | 234 → 105  236 → 107  238 → 109 | 234 → 103  236 → 105  238 → 107 | D_3_-HMPMA | 237 → 105 |
| MA II | 2-HPMA  ^13^C_3_-2-HPMA | 220 → 91  223 → 94 | 220 → 89  - | D_3_-2-HPMA | 223 → 91 |
|  | 3-HPMA  ^13^C_3_-3-HPMA | 220 → 91  223 → 94 | 220 → 89  - | ^13^C_3_-^15^N-3-HPMA | 224 → 91 |
|  | DHPMA  ^13^C_3_-DHPMA | 236 → 107  236 → 107 | 236 → 128  236 → 130 | D_7_-DHBMA | 257 → 78 |

**Calculation of the amount of the EC-specific exposure relative to the overall exposure based on the obtained concentrations of labeled and unlabeled biomarkers of exposure exemplified for glycidol (DHPMA)**

Biomarker concentrations were averaged over all 20 subjects as there were no statistically significant differences observed for the different vaping conditions. The EC-specific uptake and exposure is resembled by the amount of labeled DHPMA in the urine of vapers. The amount excreted over 48 hours was then multiplied by ten to resemble for the 10% replacement in the e-liquid yielding in average 46.6 µg DHPMA in the 20 vapers:

The total exposure is given by the sum of unlabeled and labeled DHPMA:

The average total amount of DHPMA over all 20 vapers was 586.9 µg/48h.

% EC-specific DHPMA = $\frac{Labeled DHPMA x 10}{Labeled DHPMA+Unlabeled DHPMA}$ x 100

In this case, $\frac{4.66 x 10}{4.66+589.6}$ x 100 = 7.8 % = 8 %
